# Unilateral hypoplastic kidney - a novel highly penetrant feature of familial juvenile hyperuricaemic nephropathy

**DOI:** 10.1186/1471-2369-15-76

**Published:** 2014-05-09

**Authors:** Lucy A Plumb, Matko Marlais, Agnieszka Bierzynska, Howard Martin, Kim Brugger, Stephen Abbs, Moin A Saleem

**Affiliations:** 1 Department of Paediatric Nephrology, Bristol Royal Hospital for Children, Upper Maudlin Street, Bristol BS2 8BJ, UK; 2 Academic Renal Unit, University of Bristol, Dorothy Hodgkin building level 1, Whitson Street, Bristol BS1 3NY, UK; 3 Department of Molecular Genetics, Regional Molecular Genetics Laboratories, Addenbrooke’s Hospital, Cambridge CB2 0QQ, UK; 4 Department of Paediatric Nephrology, University of Bristol, Bristol Royal Hospital for Children, Bristol BS2 8BJ, UK

**Keywords:** Hypoplasia, Familial juvenile hyperuricaemic nephropathy, Uromodulin, Hepatocyte nuclear factor-1β, Renin

## Abstract

**Background:**

Familial juvenile hyperuricaemic nephropathy is a rare inherited nephropathy with genetic heterogeneity. Categorised by genetic defect, mutations in uromodulin (*UMOD*), renin (*REN*) and hepatocyte nuclear factor-1β (HNF-1β) genes as well as linkage to chromosome 2p22.1-21 have previously been identified. Knowledge of the genetics of this phenotype has provided important clues to developmental pathways in the kidney.

**Case presentation:**

We report a novel phenotype, with the typical features of hyperuricemia and renal deterioration, but with the additional unexpected feature of unilateral renal hypoplasia. Mutation analyses of the existing known genes and genetic loci were negative indicating a new monogenic cause. Interestingly two cousins of the index case did not share the latter feature, suggesting a modifier gene effect.

**Conclusion:**

Unilateral renal hypo/aplasia is usually sporadic and relatively common, with no genetic cause to date identified. This reported pedigree reveals the possibility that a new, unknown renal developmental gene may be implicated in the FJHN phenotype.

## Background

Familial juvenile hyperuricaemic nephropathy is a rare cause of renal disease in children. It is inherited in an autosomal dominant manner and is characterised by hyperuricaemia and gout, inevitably progressing to end-stage renal failure. The pathogenesis of FJHN appears to be an impairment of urate clearance relative to creatinine (fractional excretion, FE_ur_), which may present before adolescence [1]. The ensuing hyperuricaemia often develops after puberty, and may be clinically silent or manifest as gout [2]. Histological findings on biopsy suggest a chronic non-specific tubulointerstitial nephropathy [3]. Although the exact mechanism of renal dysfunction is unclear, function gradually declines and end-stage renal disease may develop within 10-15 years [4]. We present a family with FJHN whose novel renal phenotype may help to provide further understanding of the genetics surrounding renal hypoplasia.

## Case presentation

**Figure 1:**
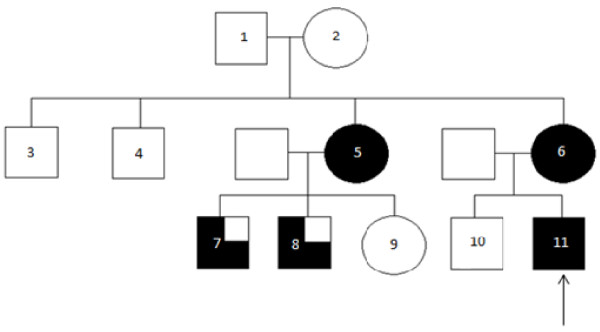
**The family pedigree studied. Arrow points to the index case.***(Partly shaded: renal impairment and hyperuricaemia/gout; fully shaded- renal impairment, hyperuricaemia/gout and unilateral renal hypoplasia).*

A 16 year old male was brought to our attention following concerns by his GP of a strong family history of renal disease. He is a fit and healthy teenager who has had no significant health problems in the past. In the family (Figure 1, Table 1) the patient’s mother had been diagnosed three years previously with renal failure requiring peritoneal dialysis and subsequent transplantation. She was noted upon further investigation to have a small right kidney on ultrasound. The patient’s maternal aunt was also in established renal failure and in addition to one small kidney on ultrasound suffered with gout. She has two sons aged 18 and 20 years with renal impairment, both with raised uric acid levels, but no evidence of renal hypoplasia. Her daughter has shown no sign of renal disease. Two maternal uncles have not had kidney problems, and neither have maternal grandparents. The patient in our case has an older unaffected brother. None of the family members have undergone a native renal biopsy.

**Table 1:** Clinical characteristics of family members

**Family member**	**Age (in 2010)**	**Most Clinical characteristics of family membersrecent creatinine/Transplant date (μmol/L)**	**Date of most recent creatinine**	**Raised uric acid level?**	**Single hypoplastic kidney?**
3	No data	No data	No data	No data	No
4	No data	No data	No data	No data	No
5	42	853	27/11/2012	Yes	Yes
6	42	Renal transplant 2006		Yes	Yes
7	20	176	3/12/2012	Yes	No
8	18	122	25/7/2012	Yes	No
9	12	68	6/7/2009	No	No
10	21	No data	No data	No	No
11	16	102	14/02/2011	Yes	Yes

Upon initial examination the patient was noted to appear well. His blood pressure of 122/55 was within normal centile range for his height and age. An ultrasound revealed a small right kidney measuring 8.2 cm which was otherwise unremarkable, as well as a normal sized although slightly echogenic left kidney, measuring 13.0 cm. The bladder and ureters were reported as normal. Initial blood tests revealed an estimated glomerular filtration rate (GFR) of 64 ml/min/1.73 m^2^, although this has since declined (table 1). Urinalysis was normal. Uric acid was initially 430 μmol/L, however on repeat testing was raised at 506 μmol/L (normal reference range 200-430 μmol/L). The patient is currently being seen at regular intervals to monitor for deterioration in renal function.

In view of the apparent autosomal dominant transmission of this phenotype, further blood samples from the affected family members were sent to test for uromodulin (*UMOD*) and hepatocyte nuclear factor-1*β* (*HNF1β*) mutations, which were negative. The index case was also screened for mutations in the renin (*REN*) gene and the 2p22.1-p21 region using exome sequencing, however, no pathological variants were found (please see supplementary data for methodology and results).

## Conclusions

FJHN is an autosomal dominant condition characterised by a hypoexcretion of urate leading to hyperuricaemia, gout and renal disease. Renal impairment is of relatively late onset, usually occurring between 15-30 years of age [4]. Progression to end stage renal impairment may develop within 10-15 years, although may be slowed by early introduction of allopurinol [5]. Ultrasound findings of note include reduction in renal size with abnormal echogenicity, and in some cases cysts have been reported [6,7].

Diagnosis of FJHN is a clinical one, however as our knowledge of this rare disease advances, so does our ability to categorise individuals based on their underlying genetic defect. There are at present three types of FJHN. Type one (FJHN1) is associated with heterozygous mutations in the uromodulin (*UMOD*) gene on chromosome 16p12.3 (MIM 191845) [3,6-9]^.^ Type two (FJHN2) is associated with mutations in the renin gene (*REN*) on chromosome 1q32 (MIM 179820) [10], and type three (FJHN3) has been mapped to 2p22.1-21 (MIM 614227) [11]. An atypical variant of FJHN, associated with diabetes and renal cysts, has been linked to mutations in *HNF-1β* on chromosome 17q12 (MIM 189907) [12].

We describe a family with familial juvenile hyperuricaemic nephropathy (FJHN) that requires our attention for several reasons. Firstly, there is the penetrant feature of a single hypoplastic kidney alongside a normal sized kidney in three out of the five affected family members. A unilateral hypoplastic kidney in a single case of FJHN has previously been described [6]. To our knowledge this is the first report to describe renal hypoplasia as a significant feature of the FJHN phenotype in one pedigree. Secondly our kindred have shown no evidence of pathological mutations in either genes or genetic loci associated with the FJHN phenotype. Two of the major genes implicated in FJHN, uromodulin (*UMOD*) and hepatocyte nuclear factor 1β (*HNF1β*), have previously been linked to a role in nephrogenesis [6,13-15]. Negative screening in this case suggests that unknown key genes may play a crucial role in the formation of the kidney as well as in the development of FJHN.

Renal hypoplasia is defined as abnormally small kidneys (greater than two standard deviations below the expected mean when correlated with age or other growth parameters) with normal morphology but reduced nephron number [16]. Whilst true diagnosis relies on histological examination, in reality the diagnosis is often made by non-invasive diagnostic tools such as ultrasound [16,17]. UK data suggests renal hypoplasia and/or dysplasia accounts for almost a third of all cases of end stage renal disease in the paediatric population [18]. Calculating the true incidence of renal hypoplasia however may be difficult owing to the fact that the terms renal dysplasia (small kidneys with tissue maldifferentiation) and renal hypoplasia are often interchangeably and incorrectly used.

Nephrourogenesis commences at four weeks of gestation, when signals from metanephric mesenchyme (MM) initiate the formation of the ureteric bud (UB) from the nearby Wolffian duct. Contact and interactions between the cell lineages subsequently lead to proliferation and epithelial transformation of the MM, which goes on to form a functional nephron. At the same time, branching of the distal UB occurs to form the mature collecting system [15,16]. The whole process is coordinated by complex interplay between various transcription, growth factors and co-factors that are referred to as renal developmental genes (RDGs). There is now growing evidence to suggest that mutations in several key RDGs may underlie the pathogenesis of renal hypoplasia, leading to aberrant interactions between the embryonic ureteric bud and the metanephric mesenchyme during fetal development [19]. Studies into cases of ‘non-syndromic’ renal hypoplasia have identified mutations in several key genes which include *PAX2, SIX2, BNP4, SALL1, UMOD and TCF2*. Unilateral renal hypoplasia has been reported as a single feature of low penetrance in a small number of studies; however extrarenal features suggestive of FJHN are lacking [13,20]. Of the aforementioned RDGs, *TCF2* and *UMOD* have been linked to HNFJ through mutation screening [3,6,8,9,12].

*TCF2* is the gene encoding for *HNF1β*, an embryonic transcription factor expressed in the liver, kidney, and pancreas [14]. Mutations in *HNF1β* have been identified in families with FJHN particularly those with atypical features such as renal cysts or other anomalies of renal development. Although the mechanism for the phenotype in these patients is unclear, it has been speculated that *HNF1β* which is expressed at an early stage in the proximal tubule may reduce transcription of human urate transporters, although this is yet to be proven [12].

*HNF1β* is known to induce *UMOD*, a gene encoding the protein uromodulin. Several uromodulin mutations have been discovered in patients with the FJHN phenotype, now categorised as FJHN type 1 [3,6-9]. Also known as Tamm-Horsfall glycoprotein, studies suggest uromodulin plays a vital role in maintaining the low water permeability and countercurrent gradient of the medullary loop [8,21]. Defects therefore lead to an increase in urinary salt and water excretion, and consequently an increase in uric acid reabsorption in the proximal tubule.

Mutations in the renin gene have been identified in three families with a FJHN phenotype. It is proposed that high expression of the mutant *REN* gene in the juxtaglomerular cells over time results in renin-angiotensin dysregulation with reduced renin synthesis, apoptosis and nephron loss leading to progressive renal failure [10]. While normal renal size has been reported in these families, mice studies have shown that complete ablation of renin-expressing cells is associated with small and morphologically abnormal kidneys, suggesting renin is necessary to maintain morphological integrity during kidney development [22].

Piret *et al.* identified a genetic region of up to 28 candidate genes on 2p22.1-21 linked to five families with FJHN which has since been termed FJHN3. It is speculated that mutations within this region may account for the majority of FJHN cases that prove negative for *UMOD*, *REN* or *HNF-1β* mutations [11]. While it is unclear how the underlying mutation leads to the resultant phenotype, a bilateral reduction in kidney size was seen in 3/16 cases that were linked the FJHN3 locus.

Returning to our index case and his family, the penetrant feature of a renal developmental anomaly in three out of five affected members may implicate an as yet unidentified developmental gene in the pathogenesis of FJHN. The genetic heterogeneity of the condition is well described, and our pedigree supports the possibility of a new, unidentified gene implicated in both the FJHN phenotype as well as the development of renal hypoplasia [3,4,9]. Other possibilities for identifying candidate genes may be suggested based on the clinical and biochemical findings known to patients with FJHN. As one of the biochemical hallmarks of the disease is hypoexcretion, it has been hypothesised that a defect in urate transport within the proximal renal tubule, where 90% of urate is reabsorbed, may be the underlying mechanism for disease [12]. So far however no significant correlation with urate transporter genes, including *URAT1* and *hUAT* has been identified [3].

What remains to be identified is the causal gene for the phenotype described in our index case and his affected family members. The overwhelming likelihood here is of a dominantly inherited single gene defect, rather than multiple genetic defects or multifactorial elements at play due to the precise inheritance seen and highly penetrant feature of a unilateral hypoplastic kidney. Interestingly, the lack of a unilateral small kidney in the two cousins of our index case, who both have exactly the same renal impairment phenotype otherwise, would imply the effect of a modifier gene to cause this feature. The genetic identification may have major implications for the commonly seen isolated hypoplastic/absent kidney phenotype in children and adults, for which no candidate gene has to date been identified. It is important to report this pedigree, as the collection of further pedigrees worldwide will help simplify identification of the responsible gene, particularly given the possibility now of complete exome sequencing for affected patients. This will also provide insight into the disease processes present in FJHN, enabling a greater understanding of how a phenotype that often presents later in life is associated with a renal developmental anomaly. Discovery of the mutation will provide an explanation to family members; will allow appropriate counselling for future generations as well as enable clinicians to implement management strategies to prevent disease progression.

## Consent

Written informed consent was obtained from the patient for publication of this Case report. A copy of the written consent is available for review by the Editor of this journal.

## Abbreviations

UMOD: Uromodulin gene; HNF1β: Hepatocyte nuclear factor-1β gene; FJHN: Familial juvenile hyperuricaemic nephropathy; GFR: Glomerular filtration rate; MM: Metanephric mesenchyme; UB: Ureteric bud; RDGs: Renal developmental genes.

## Competing interests

None declared. Genetic analysis performed by HM was supported by the Cambridge Biomedical Research Centre, and the National Institute of Health Research.

## Authors’ contributions

LAP (corresponding author) helped to design the manuscript. She researched, drafted and revised the final manuscript for submission. MM was involved in the design of the manuscript, performed data collection on the pedigree and helped to revise the manuscript for submission. AB interpreted the data from the exome sequencing analysis and helped to revise the manuscript. HM, KB and SA performed exome sequencing on DNA from the index case, and helped to redesign and revise the manuscript for re-submission. MAS created the original concept for the manuscript and critically revised the manuscript for important intellectual content. All authors have given their approval for publication of the manuscript. MAS has given overall approval for this version to be published.

## Pre-publication history

The pre-publication history for this paper can be accessed here:

http://www.biomedcentral.com/1471-2369/15/76/prepub
